# Extraction and Characterization of Novel Natural Hydroxyapatite Bioceramic by Thermal Decomposition of Waste Ostrich Bone

**DOI:** 10.1155/2020/1690178

**Published:** 2020-08-28

**Authors:** Komal Prasad Malla, Sagar Regmi, Achyut Nepal, Sitaram Bhattarai, Ram Jeewan Yadav, Shinichi Sakurai, Rameshwar Adhikari

**Affiliations:** ^1^Research Centre for Applied Science and Technology (RECAST), Tribhuvan University, Kirtipur, Kathmandu, Nepal; ^2^Central Department of Chemistry, Tribhuvan University, Kirtipur, Kathmandu, Nepal; ^3^School of Health and Allied Sciences, Pokhara University, Pokhara, Nepal; ^4^Nepal Academy of Science and Technology (NAST), Khumaltar, Lalitpur, Nepal; ^5^Department of Chemistry, Tri-Chandra Multiple Campus, Tribhuvan University, Kathmandu, Nepal; ^6^Department of Chemsitry, Prithvi Narayan Campus, Tribhuvan University, Pokhara, Nepal; ^7^Department of Biobased Materials Science, Kyoto Institute of Technology, Kyoto, Japan

## Abstract

A novel natural hydroxyapatite (HAp) bioceramic was extracted from the ostrich cortical bone by the thermal decomposition method. HAp was characterized by different analytical tools such as thermogravimetric analysis (TGA), Fourier-transform infrared spectroscopy (FTIR), X-ray diffraction (XRD) analysis, and scanning electron microscopy (SEM). Removal of organic impurities from the bone powder was confirmed by TGA analysis. FTIR spectra of HAp confirmed the presence of the major functional groups such as phosphate (PO_4_^3−^), hydroxyl (OH^−^), and carbonate (CO_3_^2−^) in the bioceramic. The XRD data revealed that the HAp was the crystalline phase obtained by calcination of the bone powder at 950°C, and the SEM analyses confirmed the typical plate-like texture of the nanosized HAp crystals.

## 1. Introduction

At the molecular level, the animal bone is composed of 30–35% organic and 65–70% inorganic components on a dry weight basis [1]. The organic part of the bone contains mainly collagen (95%) and proteins [2]. Besides these, there are other organic substances such as chondroitin sulphate and keratin sulphate along with different lipids such as phospholipids, cholesterol, fatty acids, and triglycerides [3]. The inorganic part of the bone is mainly HAp. It is a calcium/phosphate-based bioceramic which is chemically like the inorganic constituent of the bone matrix (a very complex bone tissue) with the general formula Ca_10_ (PO_4_)_6_ (OH) _2_ [4]. This ceramic is not only biocompatible, osteoconductive, noninflammatory, and nonimmunogenic but also bioactive; i.e., it has an ability to form a direct bond with living tissues and promote tissue growth [5]. Owing to these properties and chemical likeness to the mineral constituent of the bone matrix, it is a prime filler material to replace damaged bone or is a coating on implants to promote bone ingrowths into prosthetic implants and many more non-load bearing applications [6]. HAp can either be synthesized from inorganic calcium and phosphorous-based precursors or from natural biogenic resources. However, HAp synthesized from both resources is bioactive and are considered equally for *in vitro* and *in vivo* biomedical applications. The most frequently used chemical routes of synthesis are sol-gel [7], precipitation [8], coprecipitation [9], sonochemical [10], hydrothermal [11], mechanochemical [12], and microemulsion [13]. However, the major drawbacks of chemically synthesis routes are it demands a high degree of pure chemicals in addition to the lengthy procedures associated with the synthetic routes [14]. To produce biologically preferable and to avoid the lengthy procedure, biogenic resources are alternative sources for synthesis. In comparison with chemical precursors, these sources are biologically safe as no foreign chemicals are utilized and are ecofriendly and cost effective.

Although the ostrich farms are available in Nepal since 2008, their numbers are declining throughout the world. As it is not a widely available biowaste, according to Mr. C.P. Sharma, CEO of Ostrich Nepal Pvt. Ltd, this farm is producing 1000 kg meat per day and 15–20% bone waste tentatively based on the body weight (180–300 kg) of the ostrich. For sustainable development, waste should be recycled, reused, and channeled towards the production of value-added products. In this context, the ostrich (*Struthio camelus*) bone, which is the biowaste, is an economical source of HAp for hard tissue replacement in biomedical and dental applications [15].

Furthermore, HAp extracted from biogenic resources such as mammalian, avian, and fish bones, corals, seashells, and eggshells can exhibit better biological properties in comparison with the chemical sources [16]. Moreover, such an extract contains beneficial cations such as Na^+^, K^+^, Mg^+2^, Sr^+2^, Zn^+2^, and Al^+3^ or anions such as F^−^, Cl^−^, SO_4_^−2^, and CO_3_^−2^ or presence of both ions. These resources are beneficial for biomedical applications [17]. Different researchers have used various biogenic resources for the extraction of HAp. Barakat et al. [18] and Sofronia et al. [19] used bovine bones, Lu et al. [20] used pig bone and teeth, and Abdulrahman et al. [21], Wu et al. [5], and Khandelwal and Prakash [1] used wastes from eggshells. Similarly, Ferreira et al. [22] extracted from ostrich eggshells. Kongsri et al. [23] and Panda et al. [24] extracted from fish scales, and Venkatesan et al. [25] extracted from the fish bone. Furthermore, Zainon et al. [26] and Kim et al. [27] extracted from the cuttle fish bone. Chattanathan et al. [28] used the catfish bone. Piccirillo et al. [29] used the codfish bone, and Wan et al. [30] extracted from plant resources.

Alkaline hydrothermal hydrolysis process was used to decompose and dissolve organic impurities as well as fatty tissues. Two-step thermal decomposition was done in a muffle furnace (Model no: STM-8-12, Henan Sante Furnace Technology Co. Ltd. Henan, China) to eliminate the collagen and other residual organic moieties. Water-in-oil (W/O) microemulsion technique was used to control the particle size of the extracted HAp. This technique is effective to control the particle size from the micron to the nanometer range [31]. Moreover, in this method, reverse micelles are formed when an aqueous phase containing HAp nanoparticles has been dispersed as a microdroplet in an oil phase and surrounded by a surfactant molecule. Generally, the size of microdroplets is smaller and uniform which acts as a nanoreactor to control the particle size in the emulsion intermediate [13]. In addition, the literature shows that the size of the HAp controlled by using ionic and nonionic surfactants has been reported to have a nanoscale surface area [32].

Hence, the goal of this study is to synthesize natural HAp from the waste ostrich bone by applying alkaline hydrothermal hydrolysis and two-step thermal decomposition methods, followed by particle size control by applying the water–in-oil (W/O) microemulsion technique and to find how their unique properties allow for their potential use in various biomedical applications.

## 2. Materials and Methods

### 2.1. Materials

The required amount of the femur bone of a male ostrich (2-3 years old) was collected from the commercial ostrich slaughtering house. It is located in Ostrich Nepal Pvt. Ltd, Gangolya-1, Rupendehi district, the western region of Nepal. The femur bone is categorized as cortical bones which contain the highest amount of minerals per unit weight.

### 2.2. Preparation of Bone Powder

The method adopted for the extraction of HAp was the modified procedures of Barakat et al. [18] and Sobczak et al. [33]. The bone samples were cleaned to get rid of visible impurities employing a sharp knife. Then, they were cut into small pieces using a hacksaw. These pieces were boiled for about 4 h in a closed container to remove macroscopic adhering impurities. Subsequently, the samples were washed multiple times with distilled water and later immersed in acetone for 3 h to remove the invisible fat. They were then dried in a hot air oven for 12 h at 120°C to avoid shoot formation during grinding. The dried bone samples were crushed into small pieces using an iron mortar and pestle (commonly called khaal and lora which is made by a local ironsmith in Birgunj, Nepal) and then pulverized using a grinding machine. Finally, the bone powders were sieved to separate off the particle size of less than 450 *μ*m [34]. Furthermore, the alkaline hydrothermal process was applied for the removal of residual phospholipids and fatty tissues from the bone powder. The bone powder for this was treated with a highly concentrated (4N) sodium hydroxide solution with a 1 : 40 solid/liquid weight ratio and then boiled at 250°C for 6 h with continuous stirring according to the modified protocol. Finally, the bone powder was washed multiple times to neutralize the pH and then dried in a hot air oven for 3 h at 80°C to get the final yield.

### 2.3. Extraction of HAp Using Thermal Decomposition of the Bone Powder

In general, natural HAp can be extracted by calcination of the bone based on natural resources which could be a reliable and chemical-free method [35]. In this study, 100 g of the bone powder of particle sizes ranging from 0 to 450 *μ*m was placed in the calcination boat and then heated in a muffle furnace at a rate of 5°C/min from room temperature to 650°C for 6 h. The calcined samples were taken out, and then the furnace was cooled slowly to room temperature. The samples were further heated to 950°C for 6 h under a similar heating and cooling rate to get the final product, following the modified method of Sobczak et al. [33] and Barakat et al. [36].

### 2.4. Controlling Particle Size of the Extracted HAp

The sizes of the extracted HAp were not fixed. They can vary from the micrometer to the nanometer range. But in this study, the water–in-oil (W/O) reverse microemulsion technique was applied to control the size of HAp. The reagents used for the size control and reasons for choices are shown in Table 1.

The particle size of HAp was controlled using cyclohexane as an oil phase and double distilled water as a water phase. The mixtures of Triton X-100, CTAB, and TTAB were used as a nonionic and ionic surfactants in addition to the mixture of ethanol and 1-butanol which were used as a cosurfactant to stabilize the reverse microemulsion. The micelle of the reverse microemulsion formed by these mixtures which acts as a nanoreactor/capping agent only permits the entry of nanosized HAp particles inside the micelle of the reverse microemulsion and helps to isolate nanosized particles from the rest of the dispersed bulk particles in the emulsion base.

The required amount (10 g) of HAp extracted from the second step of thermal decomposition was taken in a 1L flask. The 1 : 2 v/v mixtures of distilled water and cyclohexane were transferred and agitated into the flask to prepare the microemulsion. The 1 : 1:2 v/v surfactant ratios of 2 % w/v CTAB, 10% w/v TTAB, and Triton X-100 were mixed to stabilize the microemulsion. Furthermore, 1 : 2 v/v mixtures of pure ethanol and 1-butanol were also mixed in the same emulsion as cosurfactants to enhance the activity of the surfactants and stabilization of the microemulsion. The solutions were agitated in a magnetic stirrer at 600 rpm for 2 h. At the time of agitation, there is an interparticle attraction between nano-HAp (which has a zwitter ionic behaviour) and surfactant molecules. This attraction helps them to keep it in the emulsion base and isolate the nanoparticle from the rest of the dispersed random particles of the emulsion base [37]. For better results, the emulsion was ultrasonicated at 40°C for 30 minutes. It was then centrifuged at 4500 rpm for 20 minutes to separate the emulsion-based size-controlled HAp form the rest of the solutions and oven-dried at 120^o^C for 4 h and heated again at 300^o^C for another 3 h for better results.

### 2.5. Thermogravimetric Analysis (TGA)

The mass loss pattern of the HAp powder during heating was studied via thermogravimetric analysis. A TGA analyzer, model Q600, USA, was used for this study. The TGA analysis of the HAp powder was recorded from 30°C to 1000°C at the heating rate of 10^o^C/min with a nonstop glide of nitrogen.

### 2.6. Fourier-Transform Infrared Spectroscopy (FTIR)

FTIR is the vital analytical technique for the characterization of biomaterials. To look into the presence of different functional groups, organic impurities, and the degree of probable dehydroxylation of HAp in the course of the calcination process, a Shimadzu IRT racer-100 A217064 00746, Japan, FTIR spectrophotometer was used in a transmission mode of the mid-infrared range with wavenumbers from 500 to 4000 cm^−1^ via the ATR sampling technique.

### 2.7. X-Ray Diffraction (XRD)

The particle size and crystallinity of HAp before and after size control have been investigated using the powder X-ray diffraction (XRD) technique. The XRD spectra were recorded at room temperature, using a Bruker D2, Germany, advanced X-ray diffractometer (Cu-K*α* = 1.5406 Å  radiation source) running at 40 kV and 30 mA. The diffraction profiles were accumulated over a 2*θ* range from 20° to 80° with an incremental step measurement of 0.02° using the flat plane geometry. The acquisition time was set at 2.5°/minutes for each scan. The crystallite size of the particles in the powder was calculated by using Scherer's equation.

### 2.8. Scanning Electron Microscopy (SEM)

The morphology of bone powder and the extracted HAp was analyzed using scanning electron microscopy (SEM; JEOL JSM 6490LA Analytical Scanning Electron Microscope, Japan). All the samples were sputtered with gold and then analyzed by SEM at an accelerating voltage of 10 kV.

## 3. Results and Discussion

### 3.1. Two-step Thermal Decomposition Method for the Effective Removal of Organic Moieties from Bone Powder

**Figure 1:**
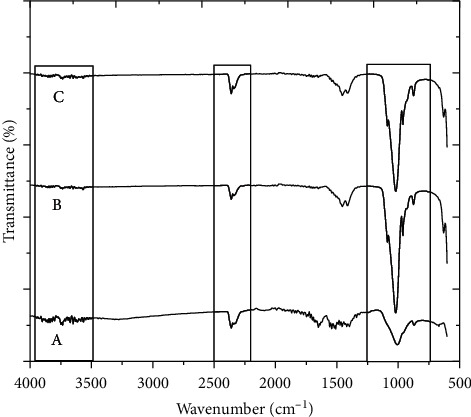
FTIR spectra showing the effect of two-step thermal decomposition on the bone powder structure: (A) raw bone powder, (B) bone powder heated at 650°C for 6 h, and (C) bone ash recalcined at 950°C for 6 h.

The FTIR spectra of the raw bone powder, before and after the thermal decomposition at 650°C and 950°C, are shown in Figure 1. This spectrum shows a series of bands in the mid-infrared (4000–500 cm^−1^) region. Different types of bonds present in various components of the bone powder give characteristic infrared absorption bands. Due to the change in the molecular environment of the bone powder, there is a shift in the intensities and position of their corresponding absorption bands [38]. The mixed broad band in the range of 3854–3568 cm^−1^ is attributed to the volatile impurities and trace amount of water molecules incorporated into the bone powder. Thermal decomposition helps to eliminate most of the volatile organic impurities from the bone powder [39]. Before heating, the hydroxyl band of HAp at 3568 cm^−1^ is not visible, and it is probably due to the overlapping of impurity bands or masking effect of other impurity bands over it [40]. The bands associated with the amide groups of proteins and collagens in the range 1315.45 cm^−1^, 1338.59 cm^−1^, 1504 cm^−1^, and 1651 cm^−1^ are clearly visible in the spectrum (Figure 1 A) due to the partial elimination of organic moieties at the time of cleaning and alkaline hydrothermal hydrolysis [27]. The band at 2360.87 cm^−1^ with a shoulder at 2337.72 cm^−1^ is credited by free carbon dioxide [41]. A strong band at 999.13 cm^−1^ is associated with P-O stretching vibration of the phosphate group [42]. A small band at 871.82 cm^−1^ is associated with the out-of-plane bending mode of a carbonate group [43], and a small band at 667.37 cm^−1^ is associated with the bending vibration of a hydroxyl group of HAp [42]. Similar types of findings have been reported by Khoo et al. in HAp extracted from the bovine bone [39].

The FTIR spectrum of the bovine bone powder reported by Barakat [36], Khoo [39], and Sobacsak [33] shows the presence of major inorganic components such as phosphate, hydroxyl, and carbonate groups and organic components such as amide-I groups from the protein constituent of the bone powder in the range 4000–500 cm^−1^. But, in this present study, comparing their results with the spectrum range 4000–500 cm^−1^ obtained for the ostrich bone ash calcined at 650°C (Figure 1 B) clearly shows that most of the organic moieties have disappeared after calcination. The spectral bands 1315.45 cm^−1^,1338.59 cm^−1^, 1396.46 cm^−1^, 1504.0 cm^−1^, and 1651.0 cm^−1^ associated with the amide-I groups of proteins and collagen which are observed in the spectrum of the raw bone powder (Figure 1 C) have totally disappeared after thermal decomposition. This significance change in the bone powder is not visible on direct heating till 950^o^C. Nevertheless, the FTIR spectra (Figure 1 B), reveal the presence of major phosphate (PO_4_^3−^), carbonate (CO_3_^2−^), and hydroxyl (OH^−^) groups. These spectra have more clearly appeared in the first-phase calcined products because the cross-linked structure in the raw bone powder is destroyed after calcination. This comparison shows that the thermal decomposition applied here is adequate to remove organic moieties from the bone powder. In addition, there are no absorption bands related to carbon-hydrogen, C-H (1396 cm^−1^), and nitrogen-hydrogen, N-H (1580 cm^−1^), bonds in the spectrum after the first-phase calcinations [39]. Therefore, all bands observed in the spectrum of Figure 1 B are associated with the inorganic components of the bone minerals. One strong and relatively broad band at 1062.78 cm^−1^, two relatively strong and sharp bands at 570.95 cm^−1^ and 603.71 cm^−1^, and another band at 956.69 cm^−1^ have appeared due to the presence of the phosphate (PO_4_^3−^) group of HAp. Bahrololoom et al. observed the similar types of two bands in the bovine cortical bone ash at 603 cm^−1^and 1051 cm^−1^ in their investigation due to the stretching vibration of the phosphate (PO_4_^3−^) group. Similarly, the bands at 871.83 cm^−1^, 1406.10 cm^−1^, and 1463.97 cm^−1^, respectively (Figure 1 B) are associated with the brushite (CaHPO_4_) and carbonate (CO_3_^2-^) groups [39]. In the bone-based HAp, the carbonate group can competitively substitute at two sites: in the hydroxyl (OH^−^) site and in the phosphate site of the HAp structure, giving A-type and B-type carbonate-substituted HAp, respectively. These two types of substitution can occur simultaneously, resulting in a mixed AB-type substitution which constitutes the mineral part of the bone. As a result, the peak position of carbonate ions in the spectra depends on whether the carbonate ions are substituted by the hydroxyl ion or the phosphate ion on the HAp lattice. There is also a relatively broad band at 3570.24 cm^−1^ (Figure 1 B) which is attributed to the hydroxyl group of HAp independently. The spectral bands of amide-I groups of proteins and collagen as mentioned above are not visible in this spectrum because of thermal decomposition which is the significance change followed by two-step calcination.

**Figure 2:**
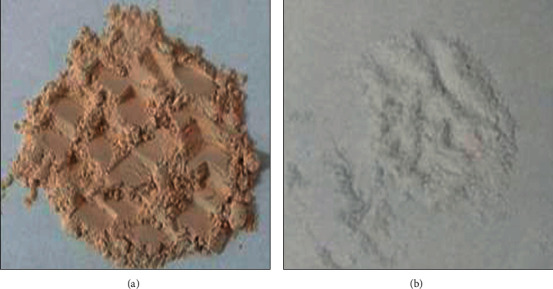
Visual observation of bone ash showing the effect of the recalcination structure: (a) bone ash calcined at 650°C for 6 h and (b) bone ash recalcined at 950°C for another 6 h.

The FTIR spectrum of the bone ash calcined again at 950°C for another 6 h is presented in Figure 1 C. This spectrum provides various spectral information indicating some changes which occurred during recalcination. The band at 2362.80 cm^−1^ has disappeared after calcination again which might be due to the elimination of residual impurities. This is in good agreement with the change in gray to clear white color of the bone ash after the second-phase calcination as shown in Figure 2.

The broad band at 3570.24 cm^−1^ observed in the spectrum of Figure 1 (A and B) has almost disappeared and been replaced by a diminutive peak at 3568.46 cm^−1^ as shown in the spectra in Figure 1 C, and this is due to the hydroxyl stretching. This spectrum is in good agreement with the spectra reported by Younesi et al. [44]. It shows the significant different between curves B and C. In this spectrum, the stretching band at 3568.46 cm^−1^ and the vibration band at 629 cm^−1^ are contributed by OH^−^ groups and the bands located at 960.55 cm^−1^, 1018.41 cm^−1^, and 1087 cm^−1^ are due to phosphate groups [20, 45]. Similarly, the bands at 1412 cm^−1^ and 1454 cm^−1^ are contributed by carbonate groups [46]. Usually, carbonate groups are the common impurity in the natural HAp extracted from animal bones [45]. Moreover, the intensity of the O-H stretching vibration in HAp is comparatively weaker than that of P-O stretching because of the HAp stoichiometry [46]. Therefore, this result shows that 6 h of heating at 950°C is the appropriate method for extraction of HAp from the bone powder after the first-phase calcination in similar conditions. In our finding, 525 g of HAp is extracted from 1 kg of the clean bone powder as a dry weight basis after two-phase calcinations at 650°C and 950°C, respectively.

### 3.2. Negligible Mass Loss of HAp Found on Heating under Inert Atmosphere

**Figure 3:**
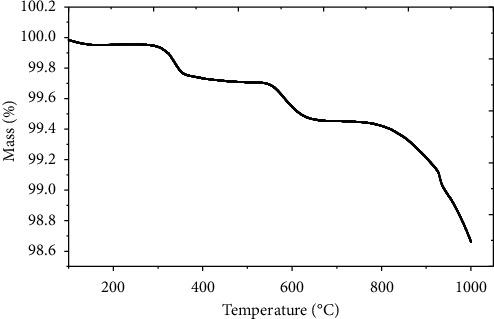
TGA curve of the HAp heated from 100°C to 1000°C under N_2_ atmosphere.

The mass loss pattern of HAp was further confirmed by TGA analysis (Figure 3). This curve indicates the three inflection points with minute mass loss on heating in an inert environment. Hu et al. investigated similar results for thermal analysis of HAp extracted from coral shells [47]. But, in this study, in the first inflection point, a negligible mass loss (0.19 wt.%) at a temperature from 275°C to 369°C corresponds to the evaporation of physisorbed and chemisorbed water molecules, as well as residual volatile organic impurities incorporated in the HAp powder [48]. Similarly, in the second inflection point, the mass loss (0.23 wt.%) at a temperature between 535°C and 646^o^C is attributed to the probable decomposition of carbonate-based impurities [21, 49]. These types of impurities are very common in bone-based resources [50].

Olsen et al. suggested that the mass loss at a temperature between 225°C and 500°C is caused by the decomposition of organic components and at a temperature higher than 500°C is a result of decomposition of the structural carbonate by release of carbon dioxide [51]. Miculescu et al. reported similar types of decomposition at a temperature between 550°C and 600°C [50]. There is no exothermic reaction related to the carbonate decomposition in the TGA curve of the bone powder investigated in this study. However, the presence of carbonate groups was also detected by FTIR analysis.

Finally, the significant mass loss (0.89 wt.%) was once found at a temperature between 770°C and 1000°C, indicating the removal of most of the residual organic moieties such as fatty tissues, collagen, chondroitin sulphate, and keratin sulphate. Furthermore, any other likely reason for continuous mass loss during heating might be due to the partial dehydroxylation of HAp in this temperature range [45, 52]. The reaction is given in the following equation:

(1) $$\begin{array}{c} {\text{Ca}}_{10}{\left({\text{PO}}_{4}\right)}_{6}{\left(\text{OH}\right)}_{2}⟶{\text{Ca}}_{10}{\left({\text{PO}}_{4}\right)}_{6}{\left(\text{OH}\right)}_{2-2x\text{O}x}+x{\text{H}}_{2}\text{O} \end{array}$$

Younesi et al. and Bahrololoom et al. investigated similar types of weight loss in their studies of HAp extracted from bovine bone ash [38, 42]. Hence, TGA studies have shown that the mass loss is due to the removal of water, residual organic moieties, and partial dehydroxylation of HAp [46].

### 3.3. Degree of Crystallinity of HAp Increased Highly after Size Control

**Figure 4:**
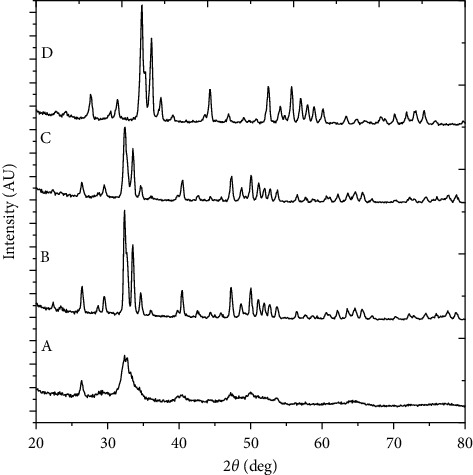
XRD patterns of (A) the raw ostrich bone powder, (B) the powder calcined at 650°C for 6 h, (C) the ash obtained after recalcination at 950°C for another 6 h, and (D) the HAp size control after the microemulsion technique.

The structural analysis of the bone powder and samples heated in different temperatures was done by XRD. The XRD profiles of the extracted HAp before and after size control are shown in Figure 4. The XRD profile of the bone powder before calcination is shown in Figure 4 A. This profile indicates the poor crystalline nature of HAp in the bone powder before calcination. The crystalline nature becomes clear on thermal decomposition at 650°C without decomposition to any other form of the calcium phosphate family. The XRD profile shown in Figure 4 B corresponds to a semicrystalline nature of HAp. This is due to the partial removal of organic impurities and presence of a slight amount of the carbonate group in the bone ash.

The diffraction profile shown in Figure 4 C is sharper in comparison with the curves in Figure 4 (A and B), which points out better crystallinity and declining order of organic impurities on increasing the calcination temperature above 650°C. The thermal decomposition peaks of HAp into alpha tricalcium phosphate (*α*-TCP) and beta tricalcium phosphate (*β*-TCP) are not observed at any temperature up to 950°C for 6 h of heating [53]. This signifies the gradual increment in the degree of sharpness of the peak or increase in the crystalline nature of HAp with the increasing calcination temperature.

The sharp and clear peak positions observed in Figure 4 D after size control confirm the phase purity and the high degree of crystallinity [43]. Venkatesan et al. also reported similar types of fully crystallized HAp extracted from the salmon fish bone [2]. Although the decomposition of HAp phases was not detected in the second-phase calcinations, dehydroxylation of HAp could have taken place after size control and reheating it up to 300°C for 3 h. Due to this reason, the peak of size-controlled HAp shifted as shown in Figure 4 D when compared to the first- and second-phase calcinated bone ash as shown in Figure 4 (B and C).

The average particle size of HAp nanoparticles calculated by Scherer's equation before and after size control is shown in Table 2.

Table 2 shows a remarkable decrement in the average particle size from 36.44 nm to 19.23 nm which points out that the reverse microemulsion is an effectual technique for controlling the particle size. The phase analysis of size-controlled HAp nanoparticles is compared with the ICCD (International Centre for Diffraction Data standard HAp) PDF card no. 00-009-0432 which shows that the major diffraction peaks at 2*θ* values of 32.026°, 33.165°, 32.424°, 49.722° 46.954°, and 34.271° corresponding to the (211), (300), (112), (213), (222), and (202) Miller planes are in good agreement with the standard HAp. Small deviation of these peaks from standard values may be due to the existence of a slight amount of foreign ions (e.g., Na^+^, K^+^, Zn^2+^, Mg^2+^, and Sr^2+^) found in this case [3]. The comparative data of diffraction profiles of prominent XRD peak position, *d-*spacing, and relative intensity which correspond to the planes (211), (300), (112), (213), (222), and (202) of standard and size-controlled HAp are shown in Table 3. It shows that the XRD profiles of HAp with the diffraction peaks obtained with *d*-spacing values of 2.79 Å, 2.76 Å, and 2.61 Å and the other *d*-spacing values match exactly with the hexagonal system with the primitive lattice. This result of XRD analysis obtained in the present investigation is in good agreement with the reported results [54].

### 3.4. Hexagonal Shape of HAp Is Clearer after Refining and Size Control

**Figure 5:**
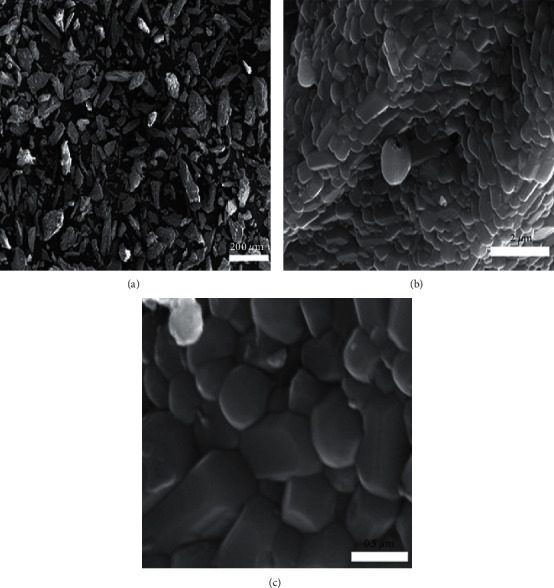
SEM micrographs of (a) the bone powder calcined at 650°C for 6 h, (b) the bone powder after recalcination at 950°C for another 6 h, and (c) the HAp obtained after the W/O microemulsion technique.

To confirm the effect of calcination and size control on the particle size and morphology, the calcined bone powder before and after size control was investigated by SEM. The SEM micrographs are shown in Figure 5. The SEM image (Figure 5(a)) for the bone powder heated at 650°C for 6 h shows a wide range of particle size and shapes. The particles have irregular shapes and size with edges and corners rather than being spherical. This irregular shape and size of the particles might be due to the grinding effect at the time of preparation. It does not show any presence of amorphous organic material, indicating that the organic moieties of the bone powder have been completely removed during calcinations. Similarly, the bone powder recalcined at 950°C for another 6 h (Figure 5(b)) shows the occurrence of a microstructural change which includes recrystallisation of the bone mineral. Bahrololoom et al. [38] attempted SEM observations of the heat-treated bovine bone and reported that the organic moieties of the bone tissue are completely eliminated during heating at 800°C. This is followed by recrystallisation of the bone mineral, i.e., HAp at 800°C, which produces a certain range of irregular shapes of crystal morphologies including spherical, hexagonal, platelet, and rod shapes. Similar types of observations were seen in this study as shown in Figure 5(b).

Moreover, the morphology of HAp particles also depends on the source of the bone, time period of calcinations, and calcination temperature [55]. However, in this study, the morphology of the particle might be influenced by the gender, age, and food habit of the ostrich from which the bone was collected. Hence, more studies are required to understand the influence of these biological factors on the morphology. Some changes in color, morphology, and recrystallisation of bone minerals due to calcinations have also been reported by Ooi et al. [55] and Sagadevan and Dakshnamoorthy [56].  Figure 5(c) shows the clear picture of the SEM micrograph of the HAp after size control. As evident, the particles have irregular shapes, including small spheres, rods, and hexagonal shape, and are also in agglomerated condition together in some parts. The size of the individual particle is in the micrometer range but has not been investigated in detail.

## 4. Conclusion

This study shows that natural HAp can easily be extracted from the raw ostrich bone powder by the two-step thermal decomposition method at temperatures 650°C and 950°C, respectively. The results showed that calcination at 650°C for 6 h produces the semidecomposed powder, while heating at 950°C for another 6 h synthesizes HAp from the raw bone powder. It can be incidental that treatment temperature and time are the key parameters in determining the composition of the extracted product. Water-in-oil (W/O) microemulsion is an efficient technique for controlling the particle size of HAp from 36.44 nm to 19.23 nm. The results of TGA, FTIR, and XRD show that the major chemical components of the prepared materials are HAp, in addition to a small amount of carbonate (CO_3_^2−^) ions which is preferable for biomedical application in bone tissue engineering. The SEM result confirms the condition of agglomeration shape, size, and nature of particles before and after size control. HAp produced from this source has a great potential to be used as a viable and economical bone graft material for non-load bearing orthopaedic application.

## Figures and Tables

**Table 1:** Reagent source and explanation for choice.

Reagents	Source	Explanation for choice
(1) Cyclohexane	Merck, Germany	Used as an oil phase. It can form reverse micelle easily with water in comparison with other organic solvents.
(2) Cetyltrimethylammonium bromide (CTAB)	Merck, Germany	Used as an ionic surfactant to produce and stabilize microemulsion.
(3) Tetradecyltrimethylammonium bromide (TTAB)	Merck, Germany	Used as an ionic surfactant to produce and stabilize microemulsion.
(4) Triton X-100 (TX-100)	Packard Co. Inc., USA	Used as the nonionic surfactant to produce and stabilize microemulsion.
(5) Ethanol (99.9%)	Merck, Germany	Used as a cosurfactant to enhance the activity of surfactants in emulsion.
(6) 1-Butanol (99.5%)	Merck, Germany	Used as a cosurfactant to enhance the activity of surfactants in emulsion.

**Table 2:** The average particle size of HAp calcined at 950°C and after size control by the reverse microemulsion technique.

HAp samples	Crystallite size in the <*h k l*> direction
Sample-C (calcined at 950°C)	36.44 nm
Sample-D (size control after calcination)	19.23 nm

**Table 3:** Comparison of phase analysis of size-controlled HAp with ICCD file no. 00-09-0432.

Miller indices (*h k l*)	*d*-spacing (Å)		2*θ* value		Relative intensity	
	Standard	Size control	Standard	Size control	Standard	Size control
211	2.79685	2.79467	31.973	34.785	100.0	100.00
300	2.69969	2.79126	33.157	33.1654	54.3	65.39
112	2.77146	2.76131	32.275	32.4240	43.7	49.61
213	1.83574	1.83373	49.620	49.7223	32.3	35.33
222	1.93385	1.93515	46.947	46.9548	24.8	33.30
202	2.62219	2.61660	34.167	34.2711	24.5	23.01

## Data Availability

The data used to support the findings of this study are available from the corresponding author upon request.
